# Hemangioma-related gene polymorphisms in the pathogenesis of intraventricular hemorrhage in preterm infants

**DOI:** 10.1007/s00381-023-05824-4

**Published:** 2023-01-19

**Authors:** Katarzyna Kosik, Dawid Szpecht, Łukasz Karbowski, Salwan R. Al-Saad, Anna Chmielarz-Czarnocińska, Marcin Minta, Anna Sowińska, Ewa Strauss

**Affiliations:** 1 Department of Neonatology, Poznan University of Medical Sciences, Polna 33 Street 60-535, Poznan, Poland; 2 Poznan University of Medical Sciences, Poznan, Poland; 3 Department of Ophthalmology, Poznan University of Medical Sciences, Poznan, Poland; 4 Department of Computer Science and Statistics, Poznan University of Medical Sciences, Poznan, Poland; 5 Institute of Human Genetics, Polish Academy of Sciences, Poznan, Poland

**Keywords:** Intraventricular hemorrhage, Gene polymorphism, Preterm neonates

## Abstract

**Purpose:**

The aim of this study was to evaluate the possible relationship between four single nucleotide polymorphisms of hemangioma-linked genes encoding for anthrax toxin receptor 1 (ANTXR1 G976A), R kinase insert domain receptor (KDR T1444C), adrenoceptor beta 2 (ADRB C79CG), and insulin-like growth factor 1 receptor (IGF-1R G3174A) and the occurrence of IVH in a population of preterm infants.

**Methods:**

The study includes a population of 105 infants born from 24 + 0 to 32 + 0 weeks of gestation and hospitalized at the Department of Neonatology (III level hospital) of Poznan University of Medical Science. Intraventricular hemorrhage was diagnosed with the use of cranial ultrasound. The classification of intraventricular bleeding was based on the Papile IVH classification.

**Results:**

The incidence of IVH was higher in infants with lower birth weight, lower APGAR scores, and low birth weight. The study revealed that IVH was approximately two times less likely to occur in infants with the allele G of IGF-1R 3174G > A.

**Conclusion:**

Identifying susceptible premature infants through genetic analysis could be a potential way to alleviate severe IVH and its subsequent consequences. Further research examining a wider range of relevant gene polymorphisms could help highlight any genetic patterns in this deleterious bleeding complication.

## Introduction

Despite declining mortality rates among premature infants, intraventricular hemorrhage (IVH) is still common among convalescents [1, 2]. IVH is believed to result from changes in the perfusion of delicate cellular structures present in the growing brain and from the immaturity of the cerebral circulatory system, which is particularly prone to hypoxic-ischemic encephalopathy [2]. Lack of blood flow causes cell death and the breakdown of the blood vessel walls, leading to bleeding.

There are well-known risk factors for IVH in infants, notably a low body weight at birth and a gestational age < 32 weeks [3]. Some other factors include the absence of prenatal steroid therapy in women at risk of premature delivery, early clamping of the umbilical cord (up to 30 s after birth), symptoms of intrauterine infection in mother and newborn, and labor and delivery complicated by bleeding or perinatal hypoxia [3–5]. Genetic factors participating in the development of IVH are still being analyzed [6]. The following genetic factors that may act as risk factors of IVH have been analyzed so far: polymorphisms of genes coding for proinflammatory cytokines [7], coagulation pathway [8], regulation of systemic blood pressure and cerebral blood flows [9], and structural components of the germinal matrix [10].

The aim of this study was to evaluate the possible relationship between changes in hemangioma-linked genes encoding for anthrax toxin receptor 1 (ANTXR1), R kinase insert domain receptor (KDR), adrenoceptor beta 2 (ADRB) and insulin-like growth factor 1 receptor (IGF-1R) and the occurrence of IVH in a population of preterm infants. The demographic data and clinical characteristics of the study group were also reviewed.

## Material and methods

### Study population

The study includes a population of 105 infants born from 24 + 0 to 32 + 0 weeks of gestation and hospitalized at the Department of Neonatology (III level hospital) of Poznan University of Medical Science. Other infants were excluded due to an increased risk of complications of prematurity by one of the following factors: birth from multiple pregnancies, birth from pregnancies complicated by the death of one of the fetuses, presence of chromosomal abnormalities, and presence of inherited errors of metabolism. Also, children are diagnosed with congenital infections from TORCH complex organisms (toxoplasmosis, other, rubella, cytomegalovirus, and herpes). All infants and their parents were Caucasian, which ensured a degree of homogeneity in the study population. Infants without antenatal steroid therapy (AST) were also excluded from the study.

### Clinical features

The following factors that may be associated with the development of IVH were studied: gender, gestational age (GA; weeks), birth weight (BW, grams); mode of delivery (vaginal birth vs cesarean section); APGAR score; pH < 7.0; blood base excess (BE) in cord blood; place of birth (inborn/outborn) and intrauterine infection (defined as a positive culture in sterile originally accompanied by clinical symptoms or pneumonia developed in 48 h after the birth).

### IVH diagnosis

Intraventricular hemorrhage was diagnosed with the use of cranial ultrasound (10 MHz transducer, Prosund α7 Premier or Aloka; 4–15 MHz transducer, MyLabEight, Esaote). Routine cranial ultrasound screenings were performed on the 1st, 3rd, and 7th days post-birth in accordance with the local hospital standards. The classification of intraventricular bleeding was based on the Papile et al. IVH classification, as shown in Table 1 [11].

**Table 1:** Ultrasound grading of intraventricular hemorrhage based on the Papile classification

**Grade**	**Papile criteria**
I	Unilateral/bilateral germinal matrix hemorrhage
II	IVH without ventricular dilatation
III	IVH with ventricular dilatation
IV	IVH extending into adjacent brain parenchyma

### Studied polymorphisms

The criteria for the selection of candidate genes in the present study were their potential involvement in the pathogenesis of IVH. We studied three single nucleotide polymorphisms (SNPs): *ANTXR1* rs119475040 (976 G > A), KDR rs34231037 (1444 T > C), and *IGF-1R* rs2229765 (3174G > A) and three mutations *FLT4* rs34255532 (2860C > T), *ADRB2* rs1042714 (79C > G), and *KDR* rs121917766 (3439C > T). Blood samples (1 ml) were taken from the patient’s peripheral vein during routine testing. Test tubes were filled with the gel containing the anticoagulant ethylenediaminetetraacetic acid dipotassium salt (K2EDTA). Then the blood was frozen and delivered to the Institute of Human Genetics of the Polish Academy of Sciences, where the genetic analysis was performed. After centrifugation for 10 min (2500 rpm), DNA was isolated from leukocytes, which was used to prepare a bank of DNA samples and determine genotypes. Studied variants were assessed using commercially available SNP tests based on TaqMan probes (Thermo Fisher Scientific, C_154335568_10; C_25612213_20; C_137540_1, C_62629284_10; C_2084765_20; C_170050247_10) employing the ABI 7900HT Fast Real-Time PCR System (Life Technologies, Carlsbad, CA).

### Ethics statement

All procedures performed in studies involving human participants were in accordance with the ethical standards of the institutional and/or national research committee and with the 1964 Helsinki declaration and its later amendments or comparable ethical standards. The study was approved by the Bioethics Committee of the Poznan University of Medical Sciences (no. 66/14 and 799/16). Informed consent was obtained from all parents.

### Statistical analysis

Statistical analysis was performed using CytelStudio version 11.1.0 (CytelStudio Software Corporation, Cambridge, MA, USA) and Statistica version 10 (Stat Soft, Inc., Tulsa, OK, USA). A *p*-value of less than 0.05 indicates statistical significance. Categorical variables were presented as a percentage. Non-normally distributed continuous variables were tested by the Shapiro–Wilk test and showed as median and ranges. To evaluate the association between prematurity complications and categorical variables, the following test were performed: the Fisher exact probability test, the chi-square test, Fisher Freeman Halton, and the chi-squared test with Yates correction. *U* Mann–Whitney test was used to analyze differences in non-normally distributed continuous variables.

## Results

The demographic and clinical characteristics of enrolled infants can be found in Table 2. In our study population, 45 (44.1%) infants developed IVH. The infants were sub-classified according to grades of IVH: 19 (18.6%) newborns were diagnosed with IVH grade I, 22 (21.56%) with grade II, 4 (3.9%) with grade III, and 0 with grade IV IVH.

**Table 2:** a Chi-square test, b Mann–Whitney test, c chi-square test with Yate’s correction, d Fisher’s exact test

	**IVH**		
	**Group without or IVH I**	**group with IVH II-IV**	***P*-value**
***Gender***			
Male	38	19	0.066^a^
Female	38	7	
***Gestational age (week)***			
< 29	32	22	0.0002^a^
≥ 29	44	4	
***Birth weight (gram)***			
< 750	8	5	0.005^a^
750–1000	17	13	
> 1000	51	8	
***Apgar score***			
1st minute	6 (1–10)	4 (1–7)	0.0002^b^
5th minute	8 (2–10)	7 (1–10)	0.003^b^
***Mode of delivery***			
Vaginal	28	12	0.487^a^
Cesarean section	48	14	
***Ph 1***	7.32 (7–7.51)	7.3 (6.9–7.44)	0.222^b^
***Be 1***	− 2.4 (− 14 to + 2.5)	− 3.1 (− 22 to − 0.6)	0.293^b^
***Intrauterine infection***			
Yes	37	22	0.002^a^
No	39	4	
***Inborn***	70	24	0.697^c^
***Outborn***	6	2	
***Deaths***			
Yes	0	1	0.255^d^
No	76	25	
***Ventilation mode***			
Noninvasive	50	5	0.00004^a^
Conventional	26	21	

The severity of IVH was inversely proportional to the gestational age. Infants born from 24 + 0 to 28 + 6 weeks of gestation were more likely to experience IVH stages II to IV as compared to those born from 29 + 0 to 32 + 0 weeks of gestation (84.61% vs 15.39%, *p* < 0.001). Similarly, infants with a lower birth weight (< 1000 g) had a higher incidence of stage II to IV intraventricular hemorrhage (75.0% vs 25.0%, *p* = 0.005). Infants in the advanced IVH group (stages II to IV) also demonstrated on average a lower Apgar score in the first and 50 min of life (6 vs 4 and 8 vs 7, respectively; *p* < 0.01).

In the case of the assessed mutations, in the studied population of infants, the absence of carriers or the presence of a single was observed; hence, changes of this type were not further investigated in relation to the development of IVH. The observed genotype and allele distribution of SNPs in infants in relation to complications of prematurity are presented in Table 3. When infants were stratified as those who did not develop IVH (60/105, 55.9%) vs those who developed IVH (stages I to IV) (45/105, 44.1%), no statistical significance was observed in terms of genetic patterns of hemangioma genes. However, when stratified as infants without IVH and stage I IVH vs infants with stage II to IV IVH, the study revealed that IVH was approximately two times less likely to occur in infants with the allele G of IGF-1R 3174G > A (OR 0.505 (0.264–0.965), *p* = 0.037). No other significant associations were found with the rest of the polymorphisms.

**Table 3:** Genotype and allele distribution of infants

**Polymorphism**	**Genotype**	**Group without IVH**	**Group with IVH I–IV**	***P*-value**	**OR**	**Group without or IVH I**	**Group with IVH II–IV**	***P*-value**	**OR**
***KDR* 1444 T > C**	Genotype								
	TT	54	42	References		71	25	References	
	CC	0	0	-	-	0	0	-	-
	TC	3	3	1.000	1.286 (0.163–10.07)	5	1	1.000	0.568 (0.012–5.467)
	Allele								
	*T*	111	87	References		147	51	References	
	*C*	3	3	0.062	0.126 (0.016–1.097)	5	1	1.000	0.577 (0.012–5.341)
***ADRB 79C* > *G***	Genotype								
	CC	20	17	References		27	10	References	
	GG	6	5	1.000	0.98 (0.198–4.653)	7	4	0.805	1.543 (0.268–7.724)
	CG	31	23	0.918	0.873 (0.347–2.208)	42	12	0.777	0.771 (0.264–2.304)
	Allele								
	*C*	71	57	References		96	32	References	
	*G*	43	33	0.994	0.956 (0.517–1.76)	56	20	0.961	1.071 (0.527–2.143)
***IGF-1R* 3174G > A**	Genotype								
	AA	15	12	References		16	11	References	
	GG	14	11	1.000	0.982 (0.286–3.364)	21	4	0.094	0.277 (0.055–1.188)
	AG	28	22	1.000	0.982 (0.346–2.817)	39	11	0.143	0.410 (0.132–1.291
	Allele								
	*A*	58	46	References		71	33	References	
	*G*	56	44	1.000	0.991 (0.549–1.787)	81	19	0.037	0.505 (0.264–0.965))

## Discussion

The aim of the current study was to explore potential genetic risk factors for the development of IVH among infants born between 24 + 0 and 32 + 0 weeks of gestation. Of the 105 infants in the study, 45, accounting for 44.1% of the study population, developed IVH, of whom 19 (18.6%) developed IVH grade I, 22 (21.56%) developed IVH grade II, and 4 (3.9%) developed IVH grade III.

The incidence of developing IVH was higher among infants who were born with a birth weight of less than 1000 g. Prematurity and low birth weight are amongst some of the well-recognized risk factors for IVH [1, 5, 12–14]. The severity of IVH in our study population was inversely proportional to gestational age, birth weight, and Apgar scores.

Furthermore, infants diagnosed with intrauterine infection had a higher risk of developing IVH compared to infants who had not been diagnosed with intrauterine infection. Wu et al. demonstrated similar findings, through which they showed that the intrauterine infection was associated with proinflammatory cytokines that can cross the placenta and blood–brain barrier to cause IVH and neonatal white matter damage [15]. It is thought that cytokines are released during intrauterine infection and lead to IVH through various mechanisms. One of the proposed mechanisms is that the inflammatory factors make the IVH worse by breaking the blood–brain barrier and increasing the rate of cerebral oxygen consumption.

In our genetic analysis of the hemangioma-related genes, infants with the allele G of the insulin-like growth factor-1 receptor (IGF-1R3174 G > A) were 50% less likely to develop moderate or severe IVH (OR: 0.5, *p* = 0.037). This gene polymorphism could potentially have a protective role in mitigating moderate or severe IVH in preterm infants. Following birth, IGF-1 circulating serum levels have been shown to decrease considerably in preterm infants as compared to full-term infants [16]. Being an important factor in early growth, reduced levels of IGF-1 in preterm infants have been associated with complications of prematurity and impaired weight gain and brain growth [17–20]. The role of IGF-1 in the brain was further highlighted in Hansen-Pupp et al. paper, showing that there is a strong correlation between the mean postnatal IGF-1 concentrations and the total volume of the brain, including the gray matter and unmyelinated white matter volume (*r* ≥ 0.44, *p* < 0.01) [21].

The effect of IGF-1 on brain complications of preterm infants has further been explored by a multicenter randomized control trial in 2020. Horsch et al. split 117 preterm infants into two groups, one of which received recombinant human IGF-1 (rhIGF-1) and IGF-binding protein-3 (rhIGFBP-3), while the second group of preterm infants received only standard of care [21]. Though not statistically significant, the results demonstrated a pattern of less incidence of IVH and IVH progression in the treated group. Similar results were reported by Ley et al., of which a nonsignificant yet notable decrease in the incidence of stages III and IV IVH was reported in a study population of 121 preterm infants, of which 61 were allocated rhIGF-1/rhIGFBP-3 [22]. The exposed group had an incidence rate of IVH stage III of roughly 13% and IVH stage IV of 8%, as compared to the control group with 23% for IVH stage III and also 23% for IVH stage IV. Further studies, with larger sample sizes, may highlight the potential role of recombinant IGF-1 as an administered drug to decrease the incidence and severity of IVH in preterm infants.

The IGF-1 pathway carries strong evidence in contributing to normal brain development in the preterm population. It is possible that the gene polymorphism of the IGF-1 receptor observed in our study (allele G of the IGF-1R 3174G > A) has an enhanced effect on the IGF-1 pathway in the brain, allowing for less incidence of moderate or severe IVH.

Genetic predisposition for IVH is continuously being explored. Genetic analysis of structural components such as fibronectin-1 polymorphisms has yielded some interesting results. A major finding was that infants with the genotype TT FN1 rs10202709 were roughly seven times (OR: 7.2, *p* < 0.045) more likely to develop stage II, III, and IV IVH compared to those with other genotypes [23]. Such findings indicate that certain fibronectin polymorphisms can have a detrimental effect on the structural integrity of the germinal matrix. Furthermore, Prasun et al. [24] found that the GG in VEGF RS1570360 and CC genotypes in VEGF RS699947 were risk factors for IVH in infants less than 28 weeks old. In another study, it was found that the genotype CC of MTHFR 1298A > C gene polymorphism increased the risk of IVH by approximately 4.5 times (OR: 4.51, *p* < 0.03) for infants less than 32 weeks of gestation. On the other hand, a genetic study of some inflammatory-related gene polymorphisms (such as those encoding for Il-1B, Il-6, TNF-alpha, and Il-1RN) [8] did not reveal any significant association with the development of IVH in preterm infants. Nonetheless, it is important to continue exploring possible associations of gene polymorphisms with IVH. Ment et al. [6] contrast these findings by stating that the actual impact of specific alleles in the development of IVH may be unknown until the whole human genome sequencing data is available [6]. They go on to state that the development of IVH is due to small but distributed genetic effects and can be due to the possible impact of causal relations of genetic targets and environmental perturbations [6].

Although the study did not evaluate ways by which to minimize the development of IVH, there is a need to examine infants born prematurely between 24 and 32 weeks to reduce the likelihood of the condition. Such examinations can be critical in reducing the severity of IVH and the mortality associated with the condition. The current study established that infants born between 24 + 0 and 28 + 6 weeks were at higher risk of developing grades I and II, whereas those born between 29 + 0 and 32 + 0 weeks of gestation were likely to develop IVH grade III. Identifying vulnerable preterm infants through genetic analysis can be a potential way forward toward mitigating severe IVH and its subsequent consequences. Further studies exploring a broader range of relevant gene polymorphisms can help highlight any genetic patterns to this detrimental hemorrhagic complication.
